# Molecular Mechanism of Pathogenesis and Treatment Strategies for AL Amyloidosis

**DOI:** 10.3390/ijms23116336

**Published:** 2022-06-06

**Authors:** Hidehiko Ikura, Jin Endo, Hiroki Kitakata, Hidenori Moriyama, Motoaki Sano, Keiichi Fukuda

**Affiliations:** Department of Cardiology, Keio University School of Medicine, 35 Shinanomachi, Shinjuku-City, Tokyo 160-8582, Japan; ikurahidehiko@keio.jp (H.I.); kitkatmed@yahoo.co.jp (H.K.); h.moriyama@keio.jp (H.M.); msano@keio.jp (M.S.); kfukuda@a2.keio.jp (K.F.)

**Keywords:** misfolded proteins, amyloid light-chain (AL) amyloidosis, monoclonal free light chains (FLCs), molecular mechanism, chemotherapy

## Abstract

In amyloid light-chain (AL) amyloidosis, small B-cell clones (mostly plasma cell clones) present in the bone marrow proliferate and secrete unstable monoclonal free light chains (FLCs), which form amyloid fibrils that deposit in the interstitial tissue, resulting in organ injury and dysfunction. AL amyloidosis progresses much faster than other types of amyloidosis, with a slight delay in diagnosis leading to a marked exacerbation of cardiomyopathy. In some cases, the resulting heart failure is so severe that chemotherapy cannot be administered, and death sometimes occurs within a few months. To date, many clinical studies have focused on therapeutics, especially chemotherapy, to treat this disease. Because it is necessary to promptly lower FLC, the causative protein of amyloid, to achieve a hematological response, various anticancer agents targeting neoplastic plasma cells are used for the treatment of this disease. In addition, many basic studies using human specimens to elucidate the pathophysiology of AL have been conducted. Gene mutations associated with AL, the characteristics of amyloidogenic LC, and the structural specificity of amyloid fibrils have been clarified. Regarding the mechanism of cellular and tissue damage, the mass effect due to amyloid deposition, as well as the toxicity of pre-fibrillar LC, is gradually being elucidated. This review outlines the pathogenesis and treatment strategies for AL amyloidosis with respect to its molecular mechanisms.

## 1. Introduction

Amyloidosis is a group of diseases characterized by the formation of β-sheet structure-rich large polymers by misfolded proteins, known as amyloids, which are deposited in target organs. More than 30 types of amyloid precursor proteins have been identified, and systemic amyloidosis has been classified based on the precursor proteins and the corresponding clinical pathology. Differentiating the major two types of cardiac amyloidosis, amyloid light-chain (AL) amyloidosis and transthyretin amyloidosis (ATTR), is important from the perspective of different treatment methods. In ATTR, transthyretin, a carrier protein produced in the liver [1,2], is destabilized into monomers and aggregates to form amyloid fibrils that are deposited in the body’s tissues, especially the heart [3]. On the other hand, in AL amyloidosis, amyloid fibrils derived from the light chain of monoclonal immunoglobulin produced by abnormal plasma cells accumulate in multiple organs such as the heart, kidney, liver, gastrointestinal tract, and peripheral nerves, causing various clinical symptoms [4,5,6,7,8]. At the time of diagnosis, more than 69% of patients already have lesions in multiple organs [9]. Cardiac involvement greatly influences survival, and elevated levels of NT-proBNP and troponin T as serum markers correlate with prognosis [10].

The treatment of AL amyloidosis aims to achieve a hematological complete response (CR) and stop the progression of organ damage. Autologous stem cell transplantation (ASCT) is the first-line treatment, but its implications should be carefully considered due to high treatment-related mortality [11,12,13]. Chemotherapy was administered to patients with AL who were ineligible for ASCT. A recent phase III study ‘ANDROMEDA’ revealed that a four-drug combination therapy (Dara-CyBorD) with cyclophosphamide, bortezomib, dexamethasone, and daratumumab (a human CD38 monoclonal antibody) provides a high rate of rapid hematological CR with high safety, resulting in sufficient organ improvement [14]. Based on the results of this trial, the US Food and Drug Administration (FDA) approved Dara-CyBorD therapy for AL amyloidosis in 2021 for the first time.

## 2. Pathogenesis of AL Amyloidosis

### 2.1. Common Mechanisms of Amyloid Fibril Formation

**Figure 1:**
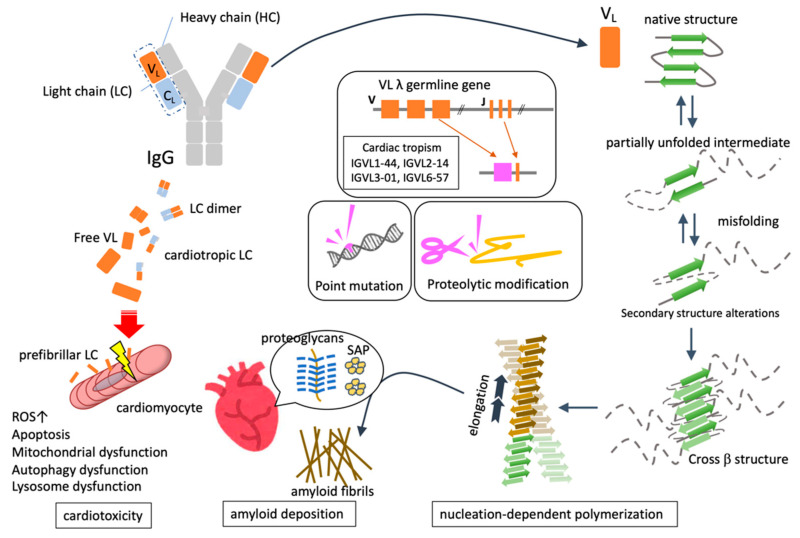
Molecular mechanisms of cardiotoxicity in AL amyloidosis.

The formation of amyloid fibrils, which is the essence of amyloidosis, requires multiple steps, such as the unfolding of precursor proteins, followed by misfolding, nucleation, polymerization, fiber elongation, and tissue deposition (Figure 1).

#### 2.1.1. Protein Unfolding and Misfolding

Proteins present a properly folded three-dimensional conformation that preserves their function and quantity. After the protein polypeptide chain is synthesized in the endoplasmic reticulum, protein folding spontaneously occurs according to the amino acid sequence, based on thermodynamic principles [15]. However, proteins are prone to transitioning into an unstable unfolding state and require chaperones to maintain the correct folding structure. Various extracellular stimuli, such as low pH, oxidation, and high temperature, disrupt the three-dimensional structure and promote the unfolding of polypeptide chains. For the amyloid fibril formation reaction, precursor proteins must present such a partially unfolded structure (so-called “partially unfolded intermediate”) [16]. Unfolded proteins usually return to their native structure naturally but are sometimes folded into a false structure that is different from the original conformation. Misfolded proteins are usually degraded and removed by the proteasome [17], but some of them are released extracellularly and reassembled into a three-dimensional conformation that is rich in β-sheets and polymerizable with each other to form amyloid fibrils [18]. 

During temporary protein unfolding, hidden hydrophobic residues are naturally exposed and proteases approach them and cleave slightly, increasing protein instability and accelerating misfolding and aggregation. As in the case of variant transthyretin amyloidosis (ATTRv), partial unfolding or misfolding may easily occur because of a change in the amino acid sequence (genomic single-point mutation) of *transthyretin* (*TTR*). It has also been pointed out that misfolding is more likely to occur due to oxidation and deamidation of amino acid side chains, chemical modification of polypeptide chains, binding of metal ions, and defects in the mechanism for controlling protein biosynthesis [19]. 

#### 2.1.2. Nucleation-Dependent Polymerization

Amyloid fibril formation proceeds in two stages: nucleation and subsequent elongation. The nucleation phase refers to the process by which the misfolded protein monomers initially assemble to form a soluble oligomer, which forms the primary nucleus of amyloid fibrils. Nucleation is unlikely to occur spontaneously because it is blocked by large energy barriers and is the rate-determining step of the entire process of fibril formation. However, once nuclei are formed, amyloid fibrils can replicate and propagate their own structure along the fiber axis by sequentially binding and growing monomers, using their terminal structure as a template. This reaction mode is called “nucleation-dependent polymerization” [20].

Regardless of the precursor protein, amyloid fibrils share common structural properties, such as non-branched fibrils with an average diameter of 7.5–10 nm and the secondary structure of cross-β-sheets [21]. Intermolecular hydrogen bonds between the amide and carbonyl groups of the main chain between the protein monomers stabilize the β-sheet structure. The β-sheet polypeptide chains adjacent to each other form a protofibril, which forms an amyloid fibril by stacking β-strands perpendicular to the fiber axis to form a cross-β structure [22]. The amyloid fibril structure exhibits an alternating combination of hydrophobic and hydrophilic parts along the fiber axis, which is why Congo red dye intercalates to show apple-green birefringence when observed under a polarizing microscope.

#### 2.1.3. Deposition of Amyloid Fibrils

The tropism of organ involvement in amyloidosis depends on the type of amyloid precursor protein; however, the mechanism is not yet understood. Even in the same type of amyloidosis, the target organs of amyloid accumulation change according to slight differences in the amino acid sequences of precursor proteins. In ATTRv, the mutation site of *TTR* strikingly affects the organs, timing of amyloid deposition, and clinical features. In addition, fragmentation of ATTR by proteolysis alters the characteristics of the target organs [23]. On the other hand, in AL, as will be described later, the gene polymorphism/amino acid sequence of the variable region of LC affects the tissue deposition pattern of amyloid fibrils. In systemic amyloidosis, nucleation and fibril formation generally depend on the concentration of the precursor proteins. Therefore, it is considered important for organ tropism that, in addition to the presence of fibril seeds, multiple conditions of the tissue environment, such as sufficient local concentration and low pH, are met at the same time. As other factors on the tissue side, the extracellular matrix (glycosaminoglycan, collagen), protease, shearing force, and metals are considered to be important in promoting aggregation and oligomer formation [24].

## 3. Characteristics of AL Amyloidosis

### 3.1. FLC as a Precursor Protein of AL

AL amyloidosis is a disease where monoclonal FLCs produced by abnormal plasma cells in the bone marrow form amyloid fibrils that are deposited in tissues. Since immunoglobulins (Ig) recognize a wide variety of antigens, each Ig is produced with a unique amino acid sequence and three-dimensional structure. Therefore, in the case of AL, the amino acid sequence and three-dimensional structure of the precursor LC in each patient are different, which results in a very diverse clinical presentation. Human Ig is a heterotetrameric glycoprotein composed of two heavy chains (HCs) and two LCs, and a single LC is composed of two domains: a variable domain (VL) involved in antigen binding and a constant domain (CL). LC belongs to either the λ or κ family and exhibits overwhelming diversity due to the random recombination of multiple gene segments.

### 3.2. Quantitative and Qualitative Abnormality of FLC

Amyloid fibril formation generally results due to quantitative abnormalities in addition to qualitative abnormalities of the precursor proteins. In AL, in addition to the qualitative abnormality regarding destabilization of the native structure based on the amino acid sequence of VL, a quantitative abnormality of overproduction of LC by abnormal plasma cells exists in the background. Although plasma cell clone proliferation is required for the production of amyloidogenic LC, amyloidogenic plasma cell clones are not abundant in the bone marrow, and plasma cell proliferation is low or difficult to detect in AL. In addition, only a few FLCs that are overproduced by neoplastic plasma cells cause amyloid formation. The fact that in patients with AL, the λ family is overrepresented 2–3 times more than the κ family [8,25] suggests that there is a gene sequence closely related to amyloid formation in the VL domain of the λ chain. Some germline genes are used in a limited manner in AL, and the IGVL6-57 (Vλ6a), IGVL3-01 (Vλ3r), and IGVL2-14 segments of the λ-chain variable region are substantially overrepresented [26]. AL is a disease in which amyloid fibrils can be deposited throughout the body, but it often shows specific deposition in important organs such as the heart and kidney. It has been pointed out that the gene sequence of some λ-chain V regions is related to this tendency. LC with the VL region derived from the rearrangement of the Vλ6a (IGVL6-57) segment is predominant in AL patients with renal disease [9], and IGVL1-44 is associated with a fivefold increase in the odds of dominant cardiac involvement [27].

### 3.3. Genomic Mutation Associated with the Pathogenesis of AL Amyloidosis

Several gene mutations associated with AL amyloidosis have been identified. It has been pointed out that pathological mutations affect the structure and function of LC, especially the VL domain, and promote amyloid aggregation. The amyloidogenic VL domain contains approximately 4–15 mutations that differ from the normal gene sequence. Although the changes that occur vary depending on the mutation site, only a few mutations contribute to the destabilization and aggregation of the VL domain toward amyloid formation or stabilization of the amyloid core structure [28,29]. Amino acid substitutions by point mutations reduce the unfolding free energy of the VL domain, leading to thermodynamic instability that promotes LC misfolding and fibril formation [30]. In addition, since the formation of the LC dimer contributes to the stabilization of LC, when a point mutation interferes with the interaction within the interface of the dimer, LC becomes monomeric and easily forms amyloids [31,32].

### 3.4. Proteolytic Modification for Amyloid Fibril Formation

Similar to other amyloid fibrils, proteolysis in AL is considered to be involved in amyloid formation because the VL domain can be observed in the fibril cores. Changes in the amino acid sequence of LC are thought to result in changes in its susceptibility to proteolysis. Several reports have shown that proteases in the blood or tissues are responsible for proteolysis. The cysteine proteases cathepsin K, L, and B co-localize with AL deposition and possess the ability to degrade AL fibrils [33,34]. Matrix metalloproteinases (MMPs) and their tissue inhibitors (TIMPs) are also highly expressed in tissues in AL [35], and the ratio of MMP-2, TIMP-1, and MMP-2/TIMP-2 is high in the serum of patients with AL [36]. It has also been reported that plasmin is required for amyloid formation in ATTR [37]; however, it conversely acts to degrade and eliminate amyloid β [38]. Hence, the role of plasmin in AL needs to be carefully evaluated. 

### 3.5. Structural Characteristics of AL Amyloid Fibril

Cryo-EM revealed the three-dimensional structures of amyloid fibrils extracted from patients with AL. The fibril structures are substantially different from the native structure of the folded VL domain, and the fibril conformation is more extended and flattened, enabling the polypeptide chain to form a single molecular fibril layer. The core structure of amyloid fibrils varies from patient to patient. Recent studies reported 77 VL domain residues (AL55) [39], 91 VL residues (λ1) [40], and 115 VL residues (FOR005) [41], showing that the number of domain residues that make up the fibril core was different. The native structure was almost completely reorganized, with parallel N to C orientations in the native state and antiparallel orientations in the fibril region around the intramolecular disulfide bond connecting the two β-sheets.

### 3.6. Deposited Components Together with Amyloid Fibril

Several components of amyloid deposits that coexist with fibril core proteins are known to modify the properties of the fibril formation process or the amyloid fibrils themselves. Serum amyloid P (SAP) is a common glycoprotein that binds to amyloid fibrils in a calcium-dependent manner. SAP itself is less susceptible to proteolysis and can confer resistance to amyloid fibrils against thermal and chemical degeneration [42]. Proteoglycans (or glycosaminoglycans), such as heparan sulfate, are another common constituent of amyloid fibrils and are thought to contribute to their development and structural stabilization in addition to affecting their localization due to co-localization with the extracellular matrix and amyloid deposits [43,44]. Clusterin, an extracellular molecular chaperone, and ApoE, which is associated with Alzheimer’s disease, are also deposited together with AL amyloid fibrils [45,46].

### 3.7. The Molecular Mechanism Underlying Cardiac Damage in AL Amyloidosis

Organ damage in amyloidosis is generally thought to be caused by the mass effects of amyloid fibril deposition. Amyloid fibrils that accumulate in large quantities in the interstitium of the tissue press on parenchymal cells, impair the coordination between cells, and harden the physical characteristics of the tissue itself. In addition, amyloid fibers interact with biological membranes to promote inflammatory reactions [47,48]. However, especially in AL, the cytotoxicity exerted by LC oligomers and aggregates before amyloid fibril formation is also an important mechanism that causes tissue damage. Although the amount of amyloid deposited in an AL heart is lower than that in ATTR, patients with AL show higher NT-proBNP levels and lower survival rates than patients with ATTR, indicating that deposited amyloid fibrils are not the only contributors to cardiac damage [49,50]. Furthermore, because the amyloid mass takes years to regress, the fact that chemotherapy decreases serum LC and subsequently improves BNP levels in patients with AL cardiomyopathy (CM) indicates that prefibrillar LC is directly toxic to the heart.

Since the severity of cardiac lesions in AL determines the prognosis, it is important to elucidate the mechanism of cardiac damage. To date, most studies elucidating the pathophysiology of AL have been conducted using human specimens, including their genomic analysis, elucidation of the features of neoplastic plasma cells in bone marrow, identification of amino acid sequences of amyloidogenic LCs, and structural analysis of amyloid fibrils. However, there is little evidence for in vivo analysis at the organ or tissue level, as there is no useful animal model that accurately mimics the disease. To date, experimental systems for assessing AL cardiotoxicity have primarily used cardiomyocytes isolated from fetal or adult rats, *C. elegans*, and zebrafish (Table 1), but there are no experiments using mammals such as adult mice, pigs, and monkeys.

*C. elegans* has no heart but is used as a model animal for heart disease because its pharynx is evolutionarily associated with the heart of vertebrates. Administration of LC extracted from patients with AL-CM induced pharyngeal dysfunction and a significant reduction in the lifespan of *C. elegans*. Cardiotropic LC induced mitochondrial dysfunction and structural abnormalities [51]. A study of LC binding partner proteins by co-immunoprecipitation and proteomics on cultured human-derived heart cells treated with LC purified from patients with AL revealed that LC binds to several intracellular proteins related to the survival and metabolism of mitochondrial proteins (mitochondrial optic atrophy-1 like protein and peroxisomal acyl-coenzyme A oxidase 1), resulting in mitochondrial damage. In another report, AL-LC-induced mitochondrial injury was caused due to high levels of ROS production, which was blocked by the administration of metal chelator or metal-binding 8-hydroxyquinoline, indicating that metal ions, especially copper ions, are strongly involved in ROS production induced by AL-LC [52].

Human AL-LC induced apoptosis in isolated adult rat cardiomyocytes by TAB-1-mediated autophosphorylation of p38-MAPK [53]. In addition, AL-LC-induced cardiac dysfunction, pericardial edema, and increased mortality improved with the administration of p38 inhibitors, showing that AL-LC-induced cardiac damage was mediated by p38 activation in zebrafish. Cardiac autophagy dysfunction triggered by lysosomal disorders based on decreased *TFEB* expression leads to cardiotoxicity of AL-LC and is restored by the administration of rapamycin in neonatal rat ventricular myocytes (NRVMs) and zebrafish [54].

Thus, studies using living organisms such as *C. elegans* or zebrafish have also shown that human cardiotropic amyloidogenic LC causes damage to their heart or heart-like organ. The experiments at cellular level in rats also show that LC contributes to cardiac dysfunction by increasing ROS production and mitochondrial damage in cardiomyocytes and partially causing apoptosis cell death. Although the p38-MAPK signaling pathway is one of the most common stress responses that may be associated with apoptosis, it has been pointed out that other stress response mechanisms such as autophagy and UPR may also be involved. There are still many unclear points regarding the molecular mechanism of myocardial injury in AL, and progress in fundamental research is an urgent issue in this field.

**Table 1:** Molecular mechanism for AL amyloidosis.

Author [Year] [Reference]	Species	Mechanisms of Myocardial Damage Directly Exerted by AL-LC
Diomede et al. [2014] [51]	*C. elegans*	Administration of LC extracted from patients with AL-CM induced a significant reduction in the lifespan and mitochondrial dysfunction
Diomede et al. [2017] [52]	*C. elegans*	LC purified from patients with severe cardiac involvement intrinsically generated high levels of ROS and when administered to *C. elegans* induced ROS production
Shi et al. [2010] [53]	Rat	Human AL-LC induced apoptosis in isolated adult rat cardiomyocytes by TAB-1-mediated autophosphorylation of p38-MAPK
Brenner et al. [2004] [55]	Rat	Human amyloid LC proteins alter cellular redox state in isolated cardiomyocytes, marked by an increase in intracellular reactive oxygen species and upregulation of the redox-sensitive protein, heme oxygenase-1
Guan et al. [2014] [54]	Rat Zebrafish	Cardiac autophagy dysfunction triggered by lysosomal disorders based on decreased TFEB expression leads to cardiotoxicity of AL-LC
Mishra et al. [2019] [56]	Zebrafish	AL-LC-induced cardiac dysfunction, pericardial edema, and increased mortality improved with the administration of p38 inhibitors

## 4. Treatment Strategies for AL Amyloidosis

Since the main cause of AL amyloidosis is amyloidogenic LC production from neoplastic plasma cells, the therapeutic goal is to rapidly suppress FLCs, achieve hematological response, and reduce organ damage. The higher the hematological response, the more likely it is that an organ response will be sustained, and 66–79% of AL patients achieving hematological CR after treatment show improvement in at least one organ and their prognosis [57]. Since autologous peripheral blood stem cell transplantation has high treatment-related mortality due to organ damage, high-dose melphalan administration with subsequent autologous stem cell transplantation (HDM/ASCT) is performed in low-risk patients after careful consideration of the implications [11,12,13,58]. In a retrospective analysis of ASCT for AL amyloidosis, the mean overall survival (OS) was 4.6 years, and the OS in patients who had survived for 1 year or more and achieved CR was over 10 years [13]. In AL patients who received ASCT, hematological CR was observed in 40% and improvement in one or more organs was observed in 66% of the patients. The usefulness of ASCT was reported in a case-control study as superior to standard chemotherapy [59]. However, patients with AL with advanced organ damage (New York Heart Association (NYHA) Classification ≥class III, cardiac Troponin T ≥0.06 ng/mL, systolic blood pressure <90, creatinine clearance <30 mL/min) at the time of diagnosis are generally ineligible for ASCT [60], and chemotherapy is administered as treatment.

**Figure 2:**
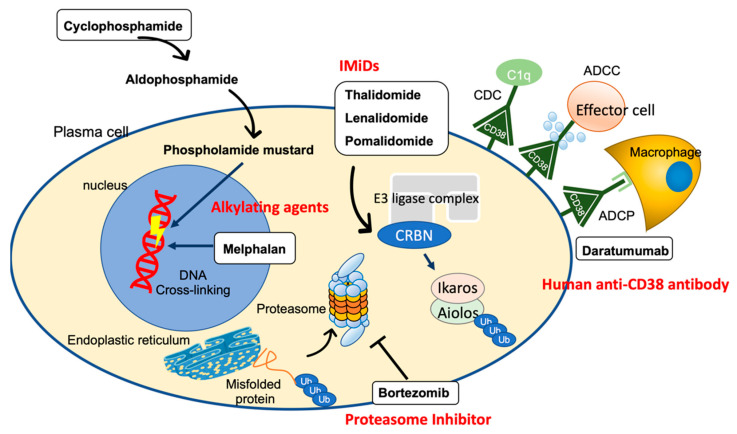
Targets and mechanisms of drugs used in AL amyloidosis.

Chemotherapeutic agents for multiple myeloma are also used for the treatment of AL because both have the same therapeutic targets for neoplastic plasma cells in the bone marrow. Oral administration of melphalan with dexamethasone (MDex) has long been the standard chemotherapy for patients ineligible for ASCT. Later, with the development of other treatments for multiple myeloma, bortezomib (a proteasome inhibitor) became the backbone of the treatment regimen and is now used in combination with MDex (BMDex) or cyclophosphamide and dexamethasone (CyBorD) [10,61,62,63]. In January 2021, the FDA approved Dara-CyBorD as a treatment for AL based on the results of a randomized, open-label, controlled phase III trial ANDROMEDA that included 388 newly diagnosed patients with AL amyloidosis (excluding patients with Mayo stage IIIB) [14]. Dara-CyBorD is the first and only FDA-approved treatment for AL amyloidosis. These advances in the effectiveness and safety of chemotherapy have led to the new concept that chemotherapy alone can be expected to provide a long-term effect. The therapeutic agents used in chemotherapy for AL amyloidosis and their mechanisms of action are described below (Figure 2).

### 4.1. Alkylating Agent

Alkylating agents such as melphalan and cyclophosphamide have multiple alkyl group sites that can be covalently attached to the nucleophilic DNA nucleotide guanine by a nucleophilic substitution reaction to form interstrand and intrastrand DNA crosslinks, which interfere with DNA replication and transcription, resulting in antineoplastic action [64]. Since alkylating agents are cell cycle non-specific antineoplastic drugs, cell reduction in normal tissues is frequently observed along with cell proliferation-active cancer cell damage. To reduce this non-specific cell injury, alkylating agents are used in combination with other agents to maximize their effectiveness at safe doses. In addition, drugs with improved cell selectivity have been developed. Notably, many drugs in clinical use, such as cyclophosphamide, are prodrugs designed to be selectively activated in tumor cells. Furthermore, alkylating agents-based hybrids with steroids that increase lipophilicity or with macrolide antibiotics possessing antitumor effects (e.g., brefeldin A and Evodiamine), and conjugates with specific peptides that are a target of the peptidase selectively overexpressed in cancer cells are developed [65,66].

#### 4.1.1. Melphalan

Melphalan, also called L-phenylalanine mustard (L-PAM), is a drug in which a phenylalanine derivative is bound to the nitrogen portion of mustard. The side effects include mucosal damage, leukopenia, and long-term administration may cause myelodysplastic syndrome and acute leukemia. It has been used as a key drug for multiple myeloma since the 1960s, and MP therapy (a combination of melphalan and prednisone) was used for the treatment of AL in the late 1970s [67]. However, the therapeutic effect of MP is poor, and MD therapy (MEL plus low-dose DEX) has long been the de facto standard treatment for AL. In a study of MD therapy administered to 96 patients, 67% had a partial hematological response (PR) or higher, and the onset of the effect was as fast as 4.5 months, while the effect on organs was observed in 48% of the patients [63]. This therapy was well tolerated, with a mean survival of 5.1 years and progression-free survival (PFS) of 3.8 years in subsequent long-term observations [68]. MEL is also used in moderate or high doses as a pre-treatment for ASCT. A randomized controlled trial of MD therapy vs. high-dose MEL-ASCT also confirmed the usefulness of MD therapy, with a median survival of 56.9 months [11]. MD therapy is effective in patients with t (11;14) [69]. However, it should be noted that regimens containing DEX may lead to volume overload in AL patients with CM, exacerbating heart failure [70].

#### 4.1.2. Cyclophosphamide

Unlike MEL, cyclophosphamide is a prodrug that acts after getting metabolized. Cyclophosphamide is first catalyzed to 4-hydroxycyclophosphamide by liver cytochrome P450 (CYP2B6, 2C9, and 3A4). 4-Hydroxycyclophosphamide interconverts with its tautomer aldophosphamide, both of which circulate and passively invade other cells. Finally, aldophosphamide is non-enzymatically metabolized to yield phosphoramide mustard, a DNA crosslinking agent with clinical significance, and acrolein, a highly reactive and toxic aldehyde species. When excreted in the urine, acrolein causes bladder mucosal cell damage, which is involved in the development of hemorrhagic cystitis and, in the long term, bladder cancer [71]. Hemorrhagic cystitis due to cyclophosphamide is common, with a frequency of 15–30% [72]. Bladder cancer occurs in 5% of patients treated with cyclophosphamide, and the incidence of bladder cancer has been reported to increase 31-fold (51-fold for <65 years old) compared to the general population [73].

### 4.2. Proteasome Inhibitors (PIs)

A misfolded protein is first repaired by a molecular chaperone such as hsp90, but if this is difficult, it is ubiquitinated by E3 ligase and degraded in the proteasome. The β1, β2, and β5 subunits of the proteasome show caspase-like/peptidylglutamyl-peptide hydrolyzing (PGPH), trypsin-like, and chymotrypsin-like activities, respectively. Bortezomib is a proteasome inhibitor with a boronic acid (BA)-containing structure. The boron element of bortezomib binds to the threonine residue of the β5 subunit, which is the active center that exhibits chymotrypsin-like activity and specifically and reversibly inhibits the proteasome. Although bortezomib is generally well tolerated when administered systemically [74], cardiac complications have been reported as side effects in addition to sensory peripheral neuropathy [75], and it is necessary to consider reducing its dose in AL patients with cardiac amyloidosis. Heart failure has mainly been reported as a cardiac adverse event (CAE) of administration of bortezomib [76,77,78], and other disorders such as conduction disorder, atrial fibrillation, and coronary artery disease have also been reported [79]. The molecular mechanism by which PIs cause myocardial injury remains unclear; however, the involvement of accumulation of misfolding proteins and mitochondrial injury in cardiomyocytes have been highlighted [80,81]. Since 2009, CyBorD has been commonly used as a front-line treatment for AL amyloidosis, including in patients with end-stage renal disease, because of its efficacy and safety [82]. It was reported that the efficacy and survival in patients treated with CyBorD were similar, even when cyclophosphamide was removed from this regimen, and dexamethasone was effective regardless of the dose. This suggests that bortezomib and low-dose dexamethasone are the main drivers of efficacy within this triplet [83].

Since special neoplastic plasma cells produce and secrete large amounts of antibodies, the endoplasmic reticulum and ubiquitin-proteasome pathways have developed to enable the synthesis, folding, and degradation of numerous proteins. As a survival strategy, plasma cells possess an advanced system of endoplasmic reticulum-associated degradation (ERAD) that excludes misfolded proteins from the ER and degrades them in the proteasome. Therefore, proteasome inhibition selectively induces excessive ER stress and apoptosis in neoplastic plasma cells [83,84].

Additionally, NF-κB activity is an important mechanism by which PIs exert anti-tumor effects. IκBα, an inhibitory protein of NF-κB, is phosphorylated and degraded by the proteasome, resulting in the activation of NF-κB. In neoplastic plasma cells, NF-κB activation enhances Bcl-2 expression to suppress apoptosis. Therefore, inhibition of the proteasome with bortezomib was initially thought to reduce the degradation of IκBα and thus not activate NF-κB, resulting in the suppression of Bcl-2 expression and promotion of apoptosis [85,86]. Paradoxically, it has also been reported that bortezomib could induce downregulation of IκBα to activate the canonical NF-κB pathway in B-cell-derived tumors [87], indicating that it is still difficult to determine the role of NF-κB in the pharmacological action of PIs.

### 4.3. Human Anti-CD38 Antibody (Daratumumab)

Daratumumab is a human IgGκ monoclonal antibody that targets the CD38 antigen expressed on the cell surface of hematopoietic tumors, including multiple myeloma and AL, and exhibits anti-tumor effects through complement-dependent cellular cytotoxicity (CDC), antibody-dependent cellular cytotoxicity (ADCC), and antibody-dependent cell-mediated phagocytosis (ADCP). In addition, the induction of apoptosis by crosslinking of antibodies, maintenance of tumor microenvironment, and increase in helper T cells by reduction of immunosuppressive cells are also considered mechanisms of action [88].

Daratumumab is generally well tolerated, and the most common side effect is the infection caused by hypoglobulinemia [89]. Intravenous administration of daratumumab requires a large volume of infusion, raising the concern of an increase in the risk of heart failure due to volume overload, making it difficult to use in patients with AL with advanced heart disease. Thereafter, a subcutaneous injection of daratumumab was developed and approved in 2020, making it safe for use in patients with AL-CM. Initially, daratumumab monotherapy was validated in critical patients with AL who received pretreatment, yet it demonstrated high efficacy in causing deep and persistent hematological and organ responses [90,91,92]. These remarkable results have paved the way for the use of daratumumab monotherapy in combination with CyBorD, the current standard therapy for AL. ANDROMEDA is a phase III study (NCT03201965) that compares two groups of CyBorD alone or in combination with the subcutaneous administration of daratumumab (Dara-CyBorD) in newly diagnosed AL patients [14]. At a median follow-up of 25.8 months, the hematologic CR rate was significantly higher in the Dara-CyBorD group than that in the CyBorD group (59.5% vs. 19.2%). Greater cardiac and renal response rates were achieved with Dara-CyBorD than with CyBorD at 18 months (cardiac: 53% vs. 24%, renal: 58% vs. 26%). Earlier and deeper hematologic responses were associated with prolonged major organ deterioration progression-free survival (MOD-PFS), and the MOD-PFS was longer in the Dara-CyBorD group than in the CyBorD group [14]. Based on these favorable results, Dara-CyBorD was approved for the first time by the FDA in January 2021 as the only treatment for AL amyloidosis.

### 4.4. Immunomodulatory Drugs (IMiDs)

Immunomodulatory drugs (IMiDs), including thalidomide and its derivatives, which bind to cereblon to exert anti-inflammatory, anti-angiogenic, and anti-tumor effects, are also used as therapeutic agents for multiple myeloma. Thalidomide was initially developed as a sedative but was found to be teratogenic and temporarily withdrawn from the market. In the 1990s, attention was once again focused on its anti-inflammatory and immunomodulatory effects, and clinical trials for multiple myeloma were conducted in 1999 to show its effectiveness. Its administration is currently approved under strict control. Cereblon forms an E3 ubiquitin ligase complex with Cullin 4 (Cul4), damage-specific DNA-binding protein 1 (DDB1), and regulator of cullins-1 (Roc1). Thalidomide or its derivatives bind to cereblon to alter its substrate specificity to ubiquitinate and degrade multiple “neo-substrates” such as Ikaros and Ailolos, exerting various pharmacological effects such as suppressing the growth of myeloma cells [93,94,95,96]. IMiDs have gained a solid position as a treatment for multiple myeloma, and several small-scale phase I and phase II trials have been conducted using IMiDs in patients with AL, showing a response rate of 41–68% [97].

### 4.5. Drugs Targeting Amyloid Fibers

With the advent of highly effective chemotherapy such as Dara-CyBorD, the time has come when AL amyloidosis can be treated. However, once the deposited amyloid does not disappear easily, it takes a very long time to achieve organ response. Therefore, patients with AL accompanied by advanced cardiac/renal disease should continue treatment for severe heart failure or maintain hemodialysis even after CR. The development of drugs that specifically target amyloid fibrils has attracted much attention and is expected to further improve the prognosis of patients with amyloidosis by preventing the deposition of amyloid fibrils and actively removing the deposited fibrils [98]. As described above, the amino acid sequence and three-dimensional structure of amyloid fibrils differ from patient to patient in AL; therefore, it is speculated that it may be difficult to obtain stable efficacy in treatments that directly target various amyloid fibrils. However, the results from the phase 1a/b study on the anti-LC amyloid fibril antibody CAEL-101 showed good organ response without causing serious adverse events. A sustained decrease in NT-proBNP levels and improvement in global longitudinal strain (GLS) were observed, notably for those with cardiac impairment [99]. Phase III trials are currently underway in untreated patients with AL amyloidosis who have advanced heart damage (Mayo Stage IIIb) [100].

## 5. Conclusions

Clinical trials are being actively conducted and useful treatments for AL amyloidosis are being developed. Currently, Dara-CyBorD is in practice as a standard regimen and is expected to have a rapid, deep hematological and organ response; however, its effectiveness in patients with severe cardiac disorder (Mayo stage IIIb) remains unclear. In addition, since the clearance of amyloid fibers is extremely slow, if a large amount of amyloid is deposited in the heart due to delayed diagnosis, strict heart failure management is required for a long period of time regardless of how successful the plasma cell treatment is. Therefore, it is increasingly important for hematologists, cardiologists, and nephrologists to work together to provide early diagnosis and treatment. Early diagnosis requires effort to share experience and knowledge among the doctors involved; however, it is expected that AI-based diagnostic support for electrocardiogram and echocardiography will become a powerful screening tool for cardiac amyloidosis in the future [101]. Using a clinical pathway as a new initiative demonstrated that any doctor could make an appropriate diagnosis quickly without much effort, reducing the severity of AL patients at the time of diagnosis [102].

To further improve the prognosis of AL cardiac amyloidosis, a therapeutic method for directly removing the deposited amyloid fibrils themselves is necessary. The development of amyloid breakers represented by antibody drugs is considered to be the next unmet clinical need. To accelerate the development of antibody drugs, it is important to continue to analyze the accurate three-dimensional structure of amyloid fibers themselves or in which the antibody binds to amyloid. As a useful model animal for evaluating the efficacy of the drug does not currently exist, the development of humanized disease model mice and its application to large experimental animals such as pigs are urgent issues. In addition, previous studies have revealed that prefibrillar LC itself in AL poses direct toxicity to the myocardium. Clarifying this detailed molecular mechanism will lead to the creation of fast-acting therapeutic agents (i.e., drugs that eliminate/block the toxic LC or cardioprotective medicine targeting specific molecules). Promotion of basic research on AL amyloidosis will become more important in the future.

## Data Availability

Not applicable.

## References

[1] Blake C.C., Geisow M.J., Oatley S.J., Rérat B., Rérat C. (1978). Structure of Prealbumin: Secondary, Tertiary and Quaternary Interactions Determined by Fourier Refinement at 1.8 A. J. Mol. Biol..

[2] Monaco H.L., Rizzi M., Coda A. (1995). Structure of a Complex of Two Plasma Proteins: Transthyretin and Retinol-Binding Protein. Science.

[3] Colon W., Kelly J.W. (1992). Partial Denaturation of Transthyretin Is Sufficient for Amyloid Fibril Formation in Vitro. Biochemistry.

[4] Brahmanandam V., McGraw S., Mirza O., Desai A.A., Farzaneh-Far A. (2014). Regression of Cardiac Amyloidosis after Stem Cell Transplantation Assessed by Cardiovascular Magnetic Resonance Imaging. Circulation.

[5] Hawkins P.N. (2002). Serum Amyloid P Component Scintigraphy for Diagnosis and Monitoring Amyloidosis. Curr. Opin. Nephrol. Hypertens..

[6] Falk R.H., Comenzo R.L., Skinner M. (1997). The Systemic Amyloidoses. N. Engl. J. Med..

[7] Merlini G., Stone M.J. (2006). Dangerous Small B-Cell Clones. Blood J. Hematol..

[8] Kyle R.A., Gertz M.A. (1995). Primary Systemic Amyloidosis: Clinical and Laboratory Features in 474 Cases. Semin. Hematol..

[9] Comenzo R.L., Zhang Y., Martinez C., Osman K., Herrera G.A. (2001). The Tropism of Organ Involvement in Primary Systemic Amyloidosis: Contributions of Ig VL Germ Line Gene Use and Clonal Plasma Cell Burden. Blood.

[10] Wechalekar A.D., Schonland S.O., Kastritis E., Gillmore J.D., Dimopoulos M.A., Lane T., Foli A., Foard D., Milani P., Rannigan L. (2013). A European Collaborative Study of Treatment Outcomes in 346 Patients with Cardiac Stage III AL Amyloidosis. Blood J. Am. Soc. Hematol..

[11] Jaccard A., Moreau P., Leblond V., Leleu X., Benboubker L., Hermine O., Recher C., Asli B., Lioure B., Royer B. (2007). High-Dose Melphalan versus Melphalan plus Dexamethasone for AL Amyloidosis. N. Engl. J. Med..

[12] Comenzo R.L., Gertz M.A. (2002). Autologous Stem Cell Transplantation for Primary Systemic Amyloidosis. Blood J. Hematol..

[13] Skinner M., Sanchorawala V., Seldin D.C., Dember L.M., Falk R.H., Berk J.L., Anderson J.J., O’Hara C., Finn K.T., Libbey C.A. (2004). High-Dose Melphalan and Autologous Stem-Cell Transplantation in Patients with AL Amyloidosis: An 8-Year Study. Ann. Intern. Med..

[14] Kastritis E., Palladini G., Minnema M.C., Wechalekar A.D., Jaccard A., Lee H.C., Sanchorawala V., Gibbs S., Mollee P., Venner C.P. (2021). Daratumumab-Based Treatment for Immunoglobulin Light-Chain Amyloidosis. N. Engl. J. Med..

[15] Dobson C.M. (2003). Protein Folding and Misfolding. Nature.

[16] Khurana R., Gillespie J.R., Talapatra A., Minert L.J., Ionescu-Zanetti C., Millett I., Fink A.L. (2001). Partially Folded Intermediates as Critical Precursors of Light Chain Amyloid Fibrils and Amorphous Aggregates. Biochemistry.

[17] Wickner S., Maurizi M.R., Gottesman S. (1999). Posttranslational Quality Control: Folding, Refolding, and Degrading Proteins. Science.

[18] Merlini G., Bellotti V. (2003). Molecular Mechanisms of Amyloidosis. N. Engl. J. Med..

[19] Ramirez-Alvarado M., Kelly J.W., Dobson C.M. (2010). Protein Misfolding Diseases: Current and Emerging Principles and Therapies.

[20] Naiki H., Gejyo F. (1999). Kinetic Analysis of Amyloid Fibril Formation. Methods in Enzymology.

[21] Eisenberg D., Jucker M. (2012). The Amyloid State of Proteins in Human Diseases. Cell.

[22] Chiti F., Dobson C.M. (2006). Protein Misfolding, Functional Amyloid, and Human Disease. Annu. Rev. Biochem..

[23] Bergström J., Gustavsson A., Hellman U., Sletten K., Murphy C.L., Weiss D.T., Solomon A., Olofsson B.-O., Westermark P. (2005). Amyloid Deposits in Transthyretin-Derived Amyloidosis: Cleaved Transthyretin Is Associated with Distinct Amyloid Morphology. J. Pathol. Bacteriol..

[24] Stevens F.J., Kisilevsky R. (2000). Immunoglobulin Light Chains, Glycosaminoglycans, and Amyloid. Cell. Mol. Life Sci..

[25] Bellotti V., Mangione P., Merlini G. (2000). Review: Immunoglobulin Light Chain Amyloidosis—The Archetype of Structural and Pathogenic Variability. J. Struct. Biol..

[26] Kourelis T.V., Dasari S., Theis J.D., Ramirez-Alvarado M., Kurtin P.J., Gertz M.A., Zeldenrust S.R., Zenka R.M., Dogan A., Dispenzieri A. (2017). Clarifying Immunoglobulin Gene Usage in Systemic and Localized Immunoglobulin Light-Chain Amyloidosis by Mass Spectrometry. Blood J. Am. Soc. Hematol..

[27] Perfetti V., Palladini G., Casarini S., Navazza V., Rognoni P., Obici L., Invernizzi R., Perlini S., Klersy C., Merlini G. (2012). The Repertoire of λ Light Chains Causing Predominant Amyloid Heart Involvement and Identification of a Preferentially Involved Germline Gene, IGLV1-44. Blood J. Am. Soc. Hematol..

[28] Blancas-Mejía L.M., Tischer A., Thompson J.R., Tai J., Wang L., Auton M., Ramirez-Alvarado M. (2014). Kinetic Control in Protein Folding for Light Chain Amyloidosis and the Differential Effects of Somatic Mutations. J. Mol. Biol..

[29] Kazman P., Vielberg M.-T., Pulido Cendales M.D., Hunziger L., Weber B., Hegenbart U., Zacharias M., Köhler R., Schönland S., Groll M. (2020). Fatal Amyloid Formation in a Patient’s Antibody Light Chain Is Caused by a Single Point Mutation. eLife.

[30] Kim Y., Wall J.S., Meyer J., Murphy C., Randolph T.W., Manning M.C., Solomon A., Carpenter J.F. (2000). Thermodynamic Modulation of Light Chain Amyloid Fibril Formation. J. Biol. Chem..

[31] DiCostanzo A.C., Thompson J.R., Peterson F.C., Volkman B.F., Ramirez-Alvarado M. (2012). Tyrosine Residues Mediate Fibril Formation in a Dynamic Light Chain Dimer Interface. J. Biol. Chem..

[32] Misra P., Blancas-Mejia L.M., Ramirez-Alvarado M. (2019). Mechanistic Insights into the Early Events in the Aggregation of Immunoglobulin Light Chains. Biochemistry.

[33] Röcken C., Stix B., Brömme D., Ansorge S., Roessner A., Bühling F. (2001). A Putative Role for Cathepsin K in Degradation of AA and AL Amyloidosis. Am. J. Pathol..

[34] Bohne S., Sletten K., Menard R., Bühling F., Vöckler S., Wrenger E., Roessner A., Röcken C. (2004). Cleavage of AL Amyloid Proteins and AL Amyloid Deposits by Cathepsins B, K, and L. J. Pathol. Bacteriol..

[35] Biolo A., Ramamurthy S., Connors L.H., O’Hara C.J., Meier-Ewert H.K., Soo Hoo P.T., Sawyer D.B., Seldin D.C., Seldin D.S., Sam F. (2008). Matrix Metalloproteinases and Their Tissue Inhibitors in Cardiac Amyloidosis: Relationship to Structural, Functional Myocardial Changes and to Light Chain Amyloid Deposition. Circulation.

[36] Tanaka K., Essick E.E., Doros G., Tanriverdi K., Connors L.H., Seldin D.C., Sam F. (2013). Circulating Matrix Metalloproteinases and Tissue Inhibitors of Metalloproteinases in Cardiac Amyloidosis. J. Am. Heart Assoc..

[37] Slamova I., Adib R., Ellmerich S., Golos M.R., Gilbertson J.A., Botcher N., Canetti D., Taylor G.W., Rendell N., Tennent G.A. (2021). Plasmin Activity Promotes Amyloid Deposition in a Transgenic Model of Human Transthyretin Amyloidosis. Nat. Commun..

[38] Tucker H.M., Kihiko M., Caldwell J.N., Wright S., Kawarabayashi T., Price D., Walker D., Scheff S., McGillis J.P., Rydel R.E. (2000). The Plasmin System Is Induced by and Degrades Amyloid-Beta Aggregates. J. Neurosci. Off. J. Soc. Neurosci..

[39] Swuec P., Lavatelli F., Tasaki M., Paissoni C., Rognoni P., Maritan M., Brambilla F., Milani P., Mauri P., Camilloni C. (2019). Cryo-EM Structure of Cardiac Amyloid Fibrils from an Immunoglobulin Light Chain AL Amyloidosis Patient. Nat. Commun..

[40] Radamaker L., Lin Y.-H., Annamalai K., Huhn S., Hegenbart U., Schönland S.O., Fritz G., Schmidt M., Fändrich M. (2019). Cryo-EM Structure of a Light Chain-Derived Amyloid Fibril from a Patient with Systemic AL Amyloidosis. Nat. Commun..

[41] Radamaker L., Karimi-Farsijani S., Andreotti G., Baur J., Neumann M., Schreiner S., Berghaus N., Motika R., Haupt C., Walther P. (2021). Role of Mutations and Post-Translational Modifications in Systemic AL Amyloidosis Studied by Cryo-EM. Nat. Commun..

[42] Tennent G.A., Lovat L.B., Pepys M.B. (1995). Serum Amyloid P Component Prevents Proteolysis of the Amyloid Fibrils of Alzheimer Disease and Systemic Amyloidosis. Proc. Natl. Acad. Sci. USA.

[43] Martin D.J., Ramirez-Alvarado M. (2011). Glycosaminoglycans Promote Fibril Formation by Amyloidogenic Immunoglobulin Light Chains through a Transient Interaction. Biophys. Chem..

[44] Blancas-Mejía L.M., Hammernik J., Marin-Argany M., Ramirez-Alvarado M. (2015). Differential Effects on Light Chain Amyloid Formation Depend on Mutations and Type of Glycosaminoglycans. J. Biol. Chem..

[45] Greene M.J., Sam F., Soo Hoo P.T., Patel R.S., Seldin D.C., Connors L.H. (2011). Evidence for a Functional Role of the Molecular Chaperone Clusterin in Amyloidotic Cardiomyopathy. Am. J. Pathol..

[46] Semba R.D., Zhang P., Zhu M., Fabbri E., Gonzalez-Freire M., Moaddel R., Geng-Spyropoulos M., Ferrucci L. (2017). A Targeted Proteomic Assay for the Measurement of Plasma Proteoforms Related to Human Aging Phenotypes. Proteomics.

[47] Berthelot K., Cullin C., Lecomte S. (2013). What Does Make an Amyloid Toxic: Morphology, Structure or Interaction with Membrane?. Biochimie.

[48] Cecchi C., Stefani M. (2013). The Amyloid-Cell Membrane System. The Interplay between the Biophysical Features of Oligomers/Fibrils and Cell Membrane Defines Amyloid Toxicity. Biophys. Chem..

[49] Merlini G., Lousada I., Ando Y., Dispenzieri A., Gertz M.A., Grogan M., Maurer M.S., Sanchorawala V., Wechalekar A., Palladini G. (2016). Rationale, Application and Clinical Qualification for NT-ProBNP as a Surrogate End Point in Pivotal Clinical Trials in Patients with AL Amyloidosis. Leukemia.

[50] Palladini G., Lavatelli F., Russo P., Perlini S., Perfetti V., Bosoni T., Obici L., Bradwell A.R., D’Eril G.M., Fogari R. (2006). Circulating Amyloidogenic Free Light Chains and Serum N-Terminal Natriuretic Peptide Type B Decrease Simultaneously in Association with Improvement of Survival in AL. Blood.

[51] Diomede L., Rognoni P., Lavatelli F., Romeo M., del Favero E., Cantù L., Ghibaudi E., di Fonzo A., Corbelli A., Fiordaliso F. (2014). A Caenorhabditis Elegans–Based Assay Recognizes Immunoglobulin Light Chains Causing Heart Amyloidosis. Blood.

[52] Diomede L., Romeo M., Rognoni P., Beeg M., Foray C., Ghibaudi E., Palladini G., Cherny R.A., Verga L., Capello G.L. (2017). Cardiac Light Chain Amyloidosis: The Role of Metal Ions in Oxidative Stress and Mitochondrial Damage. Antioxid. Redox Signal..

[53] Shi J., Guan J., Jiang B., Brenner D.A., del Monte F., Ward J.E., Connors L.H., Sawyer D.B., Semigran M.J., Macgillivray T.E. (2010). Amyloidogenic Light Chains Induce Cardiomyocyte Contractile Dysfunction and Apoptosis via a Non-Canonical P38alpha MAPK Pathway. Proc. Natl. Acad. Sci. USA.

[54] Guan J., Mishra S., Qiu Y., Shi J., Trudeau K., Las G., Liesa M., Shirihai O.S., Connors L.H., Seldin D.C. (2014). Lysosomal Dysfunction and Impaired Autophagy Underlie the Pathogenesis of Amyloidogenic Light Chain-Mediated Cardiotoxicity. EMBO Mol. Med..

[55] Brenner D.A., Jain M., Pimentel D.R., Wang B., Connors L.H., Skinner M., Apstein C.S., Liao R. (2004). Human Amyloidogenic Light Chains Directly Impair Cardiomyocyte Function through an Increase in Cellular Oxidant Stress. Circ. Res. J. Am. Heart Assoc..

[56] Mishra S., Joshi S., Ward J.E., Buys E.P., Mishra D., Mishra D., Morgado I., Fisch S., Lavatelli F., Merlini G. (2019). Zebrafish Model of Amyloid Light Chain Cardiotoxicity: Regeneration versus Degeneration. Am. J. Physiol..

[57] Martin E.B., Williams A.D., Heidel R.E., Foster J.S., Lands R.H., Kennel S.J., Wall J.S. (2018). A Functional Assay to Identify Amyloidogenic Light Chains. Amyloid.

[58] Kourelis T.V., Kumar S.K., Gertz M.A., Lacy M.Q., Buadi F.K., Hayman S.R., Zeldenrust S., Leung N., Kyle R.A., Russell S. (2013). Coexistent Multiple Myeloma or Increased Bone Marrow Plasma Cells Define Equally High-Risk Populations in Patients with Immunoglobulin Light Chain Amyloidosis. J. Clin. Oncol..

[59] Dispenzieri A., Kyle R.A., Lacy M.Q., Therneau T.M., Larson D.R., Plevak M.F., Rajkumar S.V., Fonseca R., Greipp P.R., Witzig T.E. (2004). Superior Survival in Primary Systemic Amyloidosis Patients Undergoing Peripheral Blood Stem Cell Transplantation: A Case-Control Study. Blood J. Hematol..

[60] Sher T., Dispenzieri A., Gertz M.A. (2016). Evolution of Hematopoietic Cell Transplantation for Immunoglobulin Light Chain Amyloidosis. Biol. Blood Marrow Transplant..

[61] Palladini G., Perfetti V., Obici L., Caccialanza R., Semino A., Adami F., Cavallero G., Rustichelli R., Virga G., Merlini G. (2004). Association of Melphalan and High-Dose Dexamethasone Is Effective and Well Tolerated in Patients with AL (Primary) Amyloidosis Who Are Ineligible for Stem Cell Transplantation. Blood J. Hematol..

[62] Shimazaki C., Fuchida S.-I., Suzuki K., Ishida T., Imai H., Sawamura M., Takamatsu H., Abe M., Miyamoto T., Hata H. (2016). Phase 1 Study of Bortezomib in Combination with Melphalan and Dexamethasone in Japanese Patients with Relapsed AL Amyloidosis. Int. J. Hematol..

[63] Shimazaki C., Hata H., Iida S., Ueda M., Katoh N., Sekijima Y., Ikeda S., Yazaki M., Fukushima W., Ando Y. (2018). Nationwide Survey of 741 Patients with Systemic Amyloid Light-Chain Amyloidosis in Japan. Intern. Med..

[64] Falco P., Bringhen S., Avonto I., Gay F., Morabito F., Boccadoro M., Palumbo A. (2007). Melphalan and Its Role in the Management of Patients with Multiple Myeloma. Expert Rev. Anticancer Ther..

[65] Chen Y., Jia Y., Song W., Zhang L. (2018). Therapeutic Potential of Nitrogen Mustard Based Hybrid Molecules. Front. Pharmacol..

[66] Ralhan R., Kaur J. (2007). Alkylating Agents and Cancer Therapy. Expert Opin. Ther. Pat..

[67] Kyle R.A., Greipp P.R. (1978). Primary Systemic Amyloidosis: Comparison of Melphalan and Prednisone versus Placebo. Blood J. Hematol..

[68] Palladini G., Russo P., Nuvolone M., Lavatelli F., Perfetti V., Obici L., Merlini G. (2007). Treatment with Oral Melphalan plus Dexamethasone Produces Long-Term Remissions in AL Amyloidosis. Blood J. Hematol..

[69] Sanchorawala V., Seldin D.C., Berk J.L., Sloan J.M., Doros G., Skinner M. (2010). Oral Cyclic Melphalan and Dexamethasone for Patients with AL Amyloidosis. Clin. Lymphoma Myeloma Leuk..

[70] Dhodapkar M.V., Hussein M.A., Rasmussen E., Solomon A., Larson R.A., Crowley J.J., Barlogie B. (2004). Clinical Efficacy of High-Dose Dexamethasone with Maintenance Dexamethasone/Alpha Interferon in Patients with Primary Systemic Amyloidosis: Results of United States Intergroup Trial Southwest Oncology Group (SWOG) S9628. Blood J. Hematol..

[71] Emadi A., Jones R.J., Brodsky R.A. (2009). Cyclophosphamide and Cancer: Golden Anniversary. Nat. Rev..

[72] West N.J. (1997). Prevention and Treatment of Hemorrhagic Cystitis. Pharmacother. J. Hum. Pharmacol. Drug Ther..

[73] Talar-Williams C., Hijazi Y.M., Walther M.M., Linehan W.M., Hallahan C.W., Lubensky I., Kerr G.S., Hoffman G.S., Fauci A.S., Sneller M.C. (1996). Cyclophosphamide-Induced Cystitis and Bladder Cancer in Patients with Wegener Granulomatosis. Ann. Intern. Med..

[74] Bianchi G., Ghobrial I.M. (2013). Molecular Mechanisms of Effectiveness of Novel Therapies in Multiple Myeloma. Leuk. Lymphoma.

[75] Richardson P.G., Briemberg H., Jagannath S., Wen P.Y., Barlogie B., Berenson J., Singhal S., Siegel D.S., Irwin D., Schuster M. (2006). Frequency, Characteristics, and Reversibility of Peripheral Neuropathy during Treatment of Advanced Multiple Myeloma with Bortezomib. J. Clin. Oncol..

[76] Voortman J., Giaccone G. (2006). Severe Reversible Cardiac Failure after Bortezomib Treatment Combined with Chemotherapy in a Non-Small Cell Lung Cancer Patient: A Case Report. BMC Cancer.

[77] Hacihanefioglu A., Tarkun P., Gonullu E. (2008). Acute Severe Cardiac Failure in a Myeloma Patient Due to Proteasome Inhibitor Bortezomib. Int. J. Hematol..

[78] Bockorny M., Chakravarty S., Schulman P., Bockorny B., Bona R. (2012). Severe Heart Failure after Bortezomib Treatment in a Patient with Multiple Myeloma: A Case Report and Review of the Literature. Acta Haematol..

[79] Enrico O., Gabriele B., Nadia C., Sara G., Daniele V., Giulia C., Antonio S., Mario P. (2007). Unexpected Cardiotoxicity in Haematological Bortezomib Treated Patients. Br. J. Haematol..

[80] Nowis D., Maczewski M., Mackiewicz U., Kujawa M., Ratajska A., Wieckowski M.R., Wilczyński G.M., Malinowska M., Bil J., Salwa P. (2010). Cardiotoxicity of the Anticancer Therapeutic Agent Bortezomib. Am. J. Pathol..

[81] Herrmann J., Wohlert C., Saguner A.M., Flores A., Nesbitt L.L., Chade A., Lerman L.O., Lerman A. (2013). Primary Proteasome Inhibition Results in Cardiac Dysfunction. Eur. J. Heart Fail..

[82] Palladini G., Sachchithanantham S., Milani P., Gillmore J., Foli A., Lachmann H., Basset M., Hawkins P., Merlini G., Wechalekar A.D. (2015). A European Collaborative Study of Cyclophosphamide, Bortezomib, and Dexamethasone in Upfront Treatment of Systemic AL Amyloidosis. Blood J. Am. Soc. Hematol..

[83] Adams J. (2004). The Development of Proteasome Inhibitors as Anticancer Drugs. Cancer Cell.

[84] Reimold A.M., Iwakoshi N.N., Manis J., Vallabhajosyula P., Szomolanyi-Tsuda E., Gravallese E.M., Friend D., Grusby M.J., Alt F., Glimcher L.H. (2001). Plasma Cell Differentiation Requires the Transcription Factor XBP-1. Nature.

[85] Karin M., Ben-Neriah Y. (2000). Phosphorylation Meets Ubiquitination: The Control of NF-[Kappa]B Activity. Annu. Rev. Immunol..

[86] Keats J.J., Fonseca R., Chesi M., Schop R., Baker A., Chng W.-J., van Wier S., Tiedemann R., Shi C.-X., Sebag M. (2007). Promiscuous Mutations Activate the Noncanonical NF-KappaB Pathway in Multiple Myeloma. Cancer Cell.

[87] Hideshima T., Ikeda H., Chauhan D., Okawa Y., Raje N., Podar K., Mitsiades C., Munshi N.C., Richardson P.G., Carrasco R.D. (2009). Bortezomib Induces Canonical Nuclear Factor-KappaB Activation in Multiple Myeloma Cells. Blood J. Am. Soc. Hematol..

[88] Van de Donk N.W.C.J., Janmaat M.L., Mutis T., Lammerts van Bueren J.J., Ahmadi T., Sasser A.K., Lokhorst H.M., Parren P.W.H.I. (2016). Monoclonal Antibodies Targeting CD38 in Hematological Malignancies and Beyond. Immunol. Rev..

[89] Van de Wyngaert Z., Carpentier B., Pascal L., Lionne-Huyghe P., Leduc I., Srour M., Vasseur M., Demarquette H., Terriou L., Herbaux C. (2020). Daratumumab Is Effective in the Relapsed or Refractory Systemic Light-Chain Amyloidosis but Associated with High Infection Burden in a Frail Real-Life Population. Br. J. Haematol..

[90] Sanchorawala V., Sarosiek S., Schulman A., Mistark M., Migre M.E., Cruz R., Sloan J.M., Brauneis D., Shelton A.C. (2020). Safety, Tolerability, and Response Rates of Daratumumab in Relapsed AL Amyloidosis: Results of a Phase 2 Study. Blood J. Am. Soc. Hematol..

[91] Sher T., Fenton B., Akhtar A., Gertz M.A. (2016). First Report of Safety and Efficacy of Daratumumab in 2 Cases of Advanced Immunoglobulin Light Chain Amyloidosis. Blood J. Am. Soc. Hematol..

[92] Roussel M., Merlini G., Chevret S., Arnulf B., Stoppa A.M., Perrot A., Palladini G., Karlin L., Royer B., Huart A. (2020). A Prospective Phase 2 Trial of Daratumumab in Patients with Previously Treated Systemic Light-Chain Amyloidosis. Blood J. Am. Soc. Hematol..

[93] Ito T., Ando H., Suzuki T., Ogura T., Hotta K., Imamura Y., Yamaguchi Y., Handa H. (2010). Identification of a Primary Target of Thalidomide Teratogenicity. Science.

[94] Lu G., Middleton R.E., Sun H., Naniong M., Ott C.J., Mitsiades C.S., Wong K.-K., Bradner J.E., Kaelin W.G. (2014). The Myeloma Drug Lenalidomide Promotes the Cereblon-Dependent Destruction of Ikaros Proteins. Science.

[95] Vrana J.A., Theis J.D., Dasari S., Mereuta O.M., Dispenzieri A., Zeldenrust S.R., Gertz M.A., Kurtin P.J., Grogg K.L., Dogan A. (2014). Clinical Diagnosis and Typing of Systemic Amyloidosis in Subcutaneous Fat Aspirates by Mass Spectrometry-Based Proteomics. Haematologica.

[96] Krönke J., Udeshi N.D., Narla A., Grauman P., Hurst S.N., McConkey M., Svinkina T., Heckl D., Comer E., Li X. (2014). Lenalidomide Causes Selective Degradation of IKZF1 and IKZF3 in Multiple Myeloma Cells. Science.

[97] Warsame R., LaPlant B., Kumar S.K., Laumann K., Perez Burbano G., Buadi F.K., Gertz M.A., Kyle R.A., Lacy M.Q., Dingli D. (2020). Long-Term Outcomes of IMiD-Based Trials in Patients with Immunoglobulin Light-Chain Amyloidosis: A Pooled Analysis. Blood Cancer J..

[98] Edwards C.V., Bhutani D., Mapara M., Radhakrishnan J., Shames S., Maurer M.S., Leng S., Wall J.S., Solomon A., Eisenberger A. (2019). One Year Follow up Analysis of the Phase 1a/b Study of Chimeric Fibril-Reactive Monoclonal Antibody 11-1F4 in Patients with AL Amyloidosis. Amyloid.

[99] Edwards C.V., Rao N., Bhutani D., Mapara M., Radhakrishnan J., Shames S., Maurer M.S., Leng S., Solomon A., Lentzsch S. (2021). Phase 1a/b Study of Monoclonal Antibody CAEL-101 (11-1F4) in Patients with AL Amyloidosis. Blood.

[100] Dispenzieri A., Gertz M.A., Kyle R.A., Lacy M.Q., Burritt M.F., Therneau T.M., Greipp P.R., Witzig T.E., Lust J.A., Rajkumar S.V. (2004). Serum Cardiac Troponins and N-Terminal pro-Brain Natriuretic Peptide: A Staging System for Primary Systemic Amyloidosis. J. Clin. Oncol..

[101] Goto S., Mahara K., Beussink-Nelson L., Ikura H., Katsumata Y., Endo J., Gaggin H.K., Shah S.J., Itabashi Y., MacRae C.A. (2021). Artificial Intelligence-Enabled Fully Automated Detection of Cardiac Amyloidosis Using Electrocardiograms and Echocardiograms. Nat. Commun..

[102] Brons M., Muller S.A., Rutten F.H., van der Meer M.G., Vrancken A.F.J.E., Minnema M.C., Baas A.F., Asselbergs F.W., Oerlemans M.I.F.J. (2022). Evaluation of the Cardiac Amyloidosis Clinical Pathway Implementation: A Real-World Experience. Eur. Heart J. Open.

